# RNA sequencing detects more challenging cases of somatic hypermutation in the *IgHV* gene in patients with chronic lymphocytic leukemia

**DOI:** 10.55730/1300-0144.6357

**Published:** 2026-04-26

**Authors:** Fatma ARIKAN, Pınar ATA, Şenol DEMİR, Meral ULUKÖYLÜ MENGÜÇ, Fahir ÖZKALEMKAŞ, Bedrettin ORHAN, Özgür MEHTAP, Arda BAYAR, Asu Fergün YILMAZ, Ayşe Tülin TUĞLULAR, Işık ATAGÜNDÜZ, Tayfur TOPTAŞ

**Affiliations:** 1 Division of Hematology, Department of Internal Medicine, Faculty of Medicine, Marmara University Pendik Training and Research Hospital, İstanbul, Turkiye; 2 Division of Genetics and Molecular Research, Department of Medical Genetics, Faculty of Medicine, Marmara University Pendik Training and Research Hospital, İstanbul, Turkiye; 3 Division of Hematology, Department of Internal Medicine, Faculty of Medicine, Uludağ University, Bursa, Turkiye; 4 Division of Hematology, Department of Internal Medicine, Faculty of Medicine, Kocaeli University, Kocaeli, Turkiye

**Keywords:** Chronic lymphocytic leukemia, RNA sequencing, somatic hypermutation

## Abstract

**Background/aim:**

Chronic lymphocytic leukemia (CLL) exhibits marked biological heterogeneity, and immunoglobulin heavy chain variable (*IgHV*) mutation status remains a key prognostic biomarker. We evaluated patient *IgHV* status via RNA sequencing (RNA-seq) and examined real-life clinical outcomes in a retrospective CLL cohort.

**Materials and methods:**

Eighty-seven adults diagnosed with CLL between 2020 and 2022 at three centers were included. Total RNA from peripheral blood underwent anchored multiplex polymerase chain reaction with the Immunoverse BCR-IGH IGKL v1.0 kit (Integrated DNA Technologies) and was sequenced on the MiSeq platform (Illumina). *IgHV* mutation status was determined according to ERIC 2022 guidelines, specifically regarding classification of “challenging” double-rearranged cases, with discordant productive rearrangements (one mutated, one unmutated) categorized as unmutated (u-*IgHV*) to ensure prognostic reliability. Clinical characteristics, treatment patterns, and survival outcomes were analyzed and overall survival (OS) was estimated by the Kaplan–Meier method.

**Results:**

Median age at diagnosis was 60 years and 64% of patients were male. RNA-seq identified double *IgHV* rearrangements in 73% (n = 63) of patients, 63% of whom harbored two productive rearrangements without evidence of biclonality. Unmutated *IgHV* (u-*IgHV*) was observed in 53% (n = 46). Compared to mutated *IgHV* (m-*IgHV*), patients with u-*IgHV* more frequently required first-line therapy (66% vs. 34%) and proceeded to second-line treatment more rapidly. Deletion 17p was significantly more common in the u-*IgHV* group. After median follow-up of 37.5 months, the 3-year OS rate was 91.9%, with no significant difference in OS between the m-*IgHV* and u-*IgHV* cohorts.

**Conclusion:**

RNA-seq provided detailed insight into *IgHV* mutation status and revealed a higher incidence of challenging double-rearranged cases. These real-life findings underscore the clinical utility of RNA-based *IgHV* profiling and support its use. Prospective studies comparing RNA- and DNA-based assays are warranted to validate concordance and inform risk-adapted management strategies in the treatment of CLL.

## 1. Introduction

Chronic lymphocytic leukemia (CLL) is the most common adult leukemia, comprising 25%–30% of all cases. It is a heterogeneous disease with variation in biology, clinical outcome, and prognosis. Advances in understanding its pathogenesis have led to the development of prognostic markers and targeted therapies. CLL pathogenesis is closely linked to the B-cell receptor (BCR), composed of immunoglobulin molecules and CD79a/b subunits [1]. The mutation status of the immunoglobulin heavy chain variable region (*IgHV*) gene, a key component of the BCR, allows for the classification of CLL into mutated (m-*IgHV*) and unmutated (u-*IgHV*) subtypes. Mutations with greater than 2% difference from the germline define m-*IgHV*, while a difference of less than 2% indicates u-*IgHV* [2]. Beyond these standard classifications, the European Research Initiative on CLL (ERIC) 2022 updates highlighted “challenging” cases that complicate clinical interpretation. These cases are primarily characterized by the presence of multiple productive *IgHV* rearrangements (double or triple) [3]. A critical diagnostic challenge arises in “discordant” cases, where mutated and unmutated clones coexist, requiring specific algorithmic approaches to assign a final prognostic status. Somatic hypermutation (SHM) assessment in CLL has traditionally relied on polymerase chain reaction (PCR) and Sanger sequencing as the gold standard. However, this approach is inherently complex and technically demanding; consequently, interpretation becomes particularly challenging in cases involving *IgHV* rearrangement [4]. Although Sanger sequencing remains a widely used tool, it often struggles to resolve these complex clonal architectures. Next-generation sequencing (NGS)-based assays have recently been validated as highly effective alternatives to Sanger sequencing for detecting SHM, successfully overcoming the limitations of conventional methods in resolving complex clonal backgrounds [3,5]. However, NGS also entails the challenge of determining the overall mutation status when both mutated and unmutated clones are detected [6]. RNA-based classification addresses the limitations of Sanger sequencing and NGS as it does not require the isolation of a specific clone and can better represent the disease’s biological characteristics [7].

In this study, we aimed to evaluate the applicability and diagnostic utility of RNA sequencing for determining *IgHV* mutation status in a real-life cohort, with a particular focus on its capacity to resolve diagnostically challenging cases involving double rearrangements.

## 2. Materials and methods

### 2.1. Patient cohort and sample collection

This multicenter retrospective study included 87 patients aged ≥18 years diagnosed with CLL according to the International Workshop on CLL criteria (iwCLL)**[8]**. Patients were followed between 2020 and 2022. *IgHV* mutation analysis results were retrospectively collected from patients whose peripheral blood samples were sent to the genetic laboratory from three separate university hospitals for routine testing. The analyses were performed within the framework of national health insurance reimbursement coverage. The study was conducted in accordance with the Declaration of Helsinki and approved by the local ethics committee (Approval No: 09.2023.570). Samples were submitted at the discretion of the treating physicians as part of routine clinical practice at diagnosis, prior to treatment initiation, or during follow-up. Sampling was performed when clinically indicated to evaluate the patient’s disease status accurately. Blood samples were collected in EDTA tubes and total RNA was extracted as quickly as possible using the GeneAll Hybrid-R Blood RNA Kit (GeneAll Biotechnology, Seoul, Korea), following the manufacturer’s protocol.

### 2.2. Library preparation and sequencing

Following RNA isolation, target gene regions were amplified to study rearrangements in gene segments. Library preparation began with the assessment of RNA quality and quantity. In the next step, target region clones were amplified using the Immunoverse BCR IGH IGKL v1.0 kit (Integrated DNA Technologies, Boulder, CO, USA), which employs anchored multiplex PCR (AMP) for target-enriched library construction suitable for NGS. AMP utilizes unidirectional gene-specific primers for enriching regions of interest regardless of mutation status. Lyophilized kits specifically bind to V(D)J regions to amplify rearrangement sites, which are then sequenced on an NGS platform. Molecular barcode adapters with specific molecular identifiers were applied to each molecule for accurate clonotype differentiation during data analysis. Using the immune repertoire pipeline system, clones with even single nucleotide variations can be distinguished based on sequence differences.

After library preparation, equimolar concentrations of the libraries were pooled, denatured with 0.2 N NaOH, and normalized with 200 mM Tris, and were then diluted to 10 pM for sequencing on the MiSeq platform (Illumina, San Diego, CA, USA). Open-ended amplification and V(D)J region sequencing provide more accurate coverage of the CDR3 region. Sequencing was performed using the Illumina V3 600-cycle kit, producing raw data in the FASTQ format.

### 2.3. Bioinformatic processing and mutation calling

Bioinformatic analysis and variant calling were performed with Archer Analysis Software (Integrated DNA Technologies, Inc., Coralville, IA, USA), which employs AMP chemistry for error correction, deduplication, and high-confidence alignment and mutation detection. The demultiplexed FASTQ files were processed for clonotype calling and results were provided in CSV and other formats.

**Figure 1:**
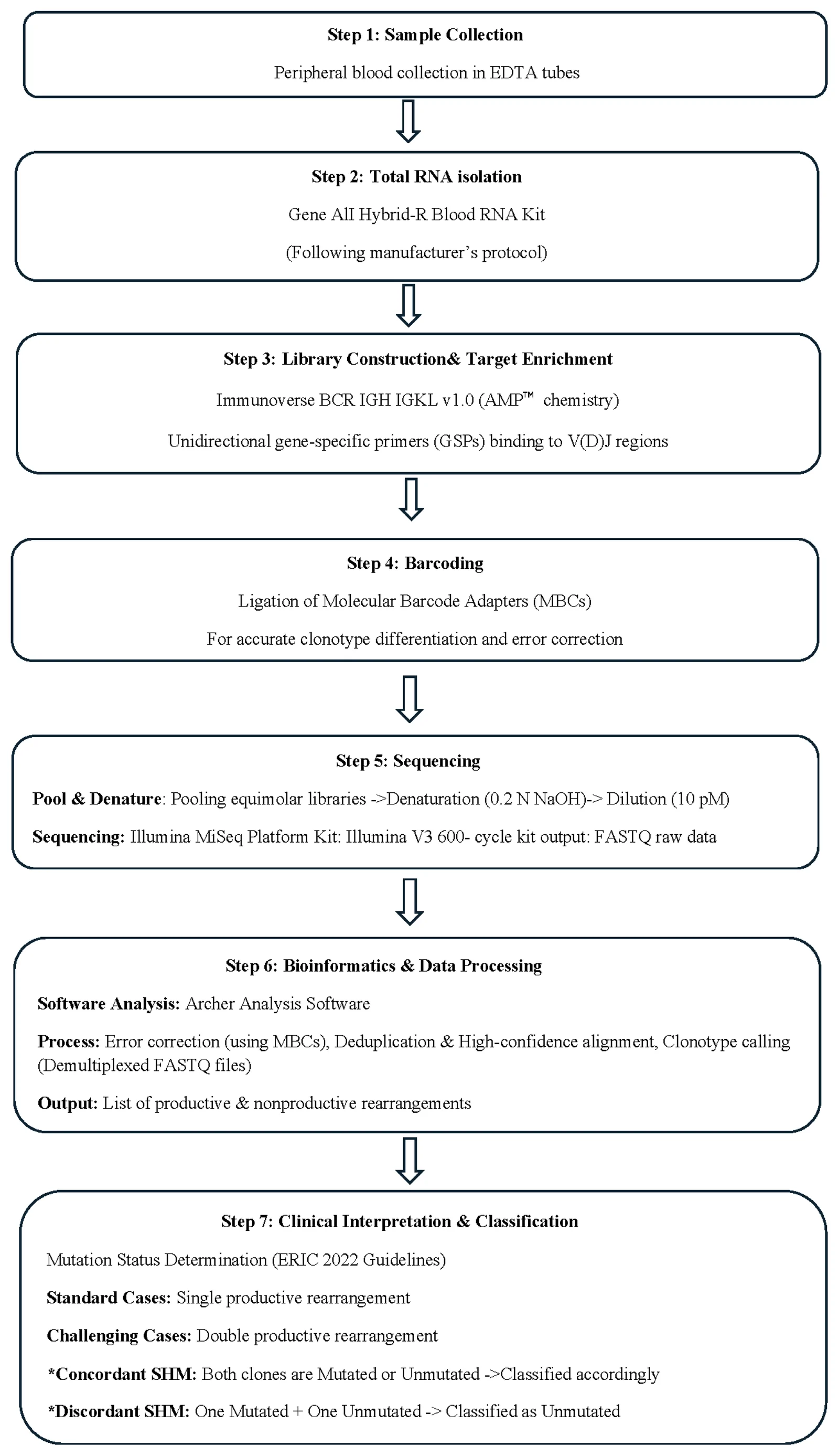
Schematic workflow of the RNA sequencing process for *IgHV* mutation analysis. Total RNA was isolated from peripheral blood samples. Libraries were prepared using the Immunoverse BCR IGH IGKL v1.0 kit employing anchored multiplex PCR (AMP) technology with molecular barcode adapters for error correction. Sequencing was performed on the Illumina MiSeq platform. Bioinformatic analysis and variant calling were conducted using Archer Analysis Software. *IgHV* mutation status was classified according to the ERIC 2022 guidelines, with specific algorithms applied for double productive rearrangements.

Interpretation was done as described in the ERIC 2022 guidelines. In standard cases, CLL mutational status was determined by productive rearrangements. For cases with one productive and one nonproductive *IgHV* rearrangement, the productive rearrangement was used. In cases with double productive rearrangements, SHM status served to determine the classification: concordant SHM was categorized as mutated or unmutated, while discordant SHM was classified as u-*IgHV* to ensure reliability [3]. The complete experimental workflow and the decision algorithm are summarized in Figure 1. This process involves amplifying and performing a sequence analysis of the entire variable heavy chain complementarity-determining region 3 (VH CDR3) to ensure comprehensive evaluation of mutations [9].

### 2.4. Clinical and cytogenetic data collection

Demographic and clinical variables were abstracted from electronic medical records at the participating centers. Cytogenetic abnormalities, including deletion 17p, deletion 13q, deletion 11q, and trisomy 12, were evaluated using interphase fluorescence in situ hybridization according to standard institutional protocols. Calculation of the International Prognostic Index for CLL (CLL-IPI) and del(17p) status were derived from the cytogenetic analysis performed at the time of diagnosis or immediately prior to first-line therapy, representing the baseline risk status of the patients.

### 2.5. Statistical analysis

Patient characteristics were reported for the m-*IgHV* and u-*IgHV* subgroups and for the entire cohort. The normality of numerical data was cross-verified using Shapiro–Wilk, Kolmogorov–Smirnov, and Anderson–Darling tests. The multitest sensitivity approach was adopted to rigorously confirm distributional assumptions, ensuring the most appropriate selection between parametric and nonparametric methods for our cohort. Differences between groups were analyzed using the Pearson chi-square, Fisher exact, or Fisher–Freeman–Halton test as appropriate. For continuous variables, group comparisons were made using the independent samples t-test for normally distributed data and the Mann–Whitney U test for nonnormally distributed data. Survival analysis for CLL patients was performed using the Kaplan–Meier method, with overall survival (OS) defined as the time from diagnosis to death or last contact. The period until the need for subsequent treatment was termed “time to next treatment” (TTNT). Multivariate prognostic evaluation was conducted using a Cox proportional hazards regression model to calculate hazards ratios (HRs) and 95% confidence intervals (CIs). The model included *IgHV* mutation status, del(17p), and CLL-IPI score as covariates. All Cox regression analyses were performed using R (version 4.3.0) software (R Foundation, Vienna, Austria). Statistical analyses were conducted using open-source Jamovi (Version 2.3.28) and JASP (Version 0.17.3) software, with significance accepted at p < 0.05.

## 3. Results

### 3.1. Patient characteristics

**Figure 2:**
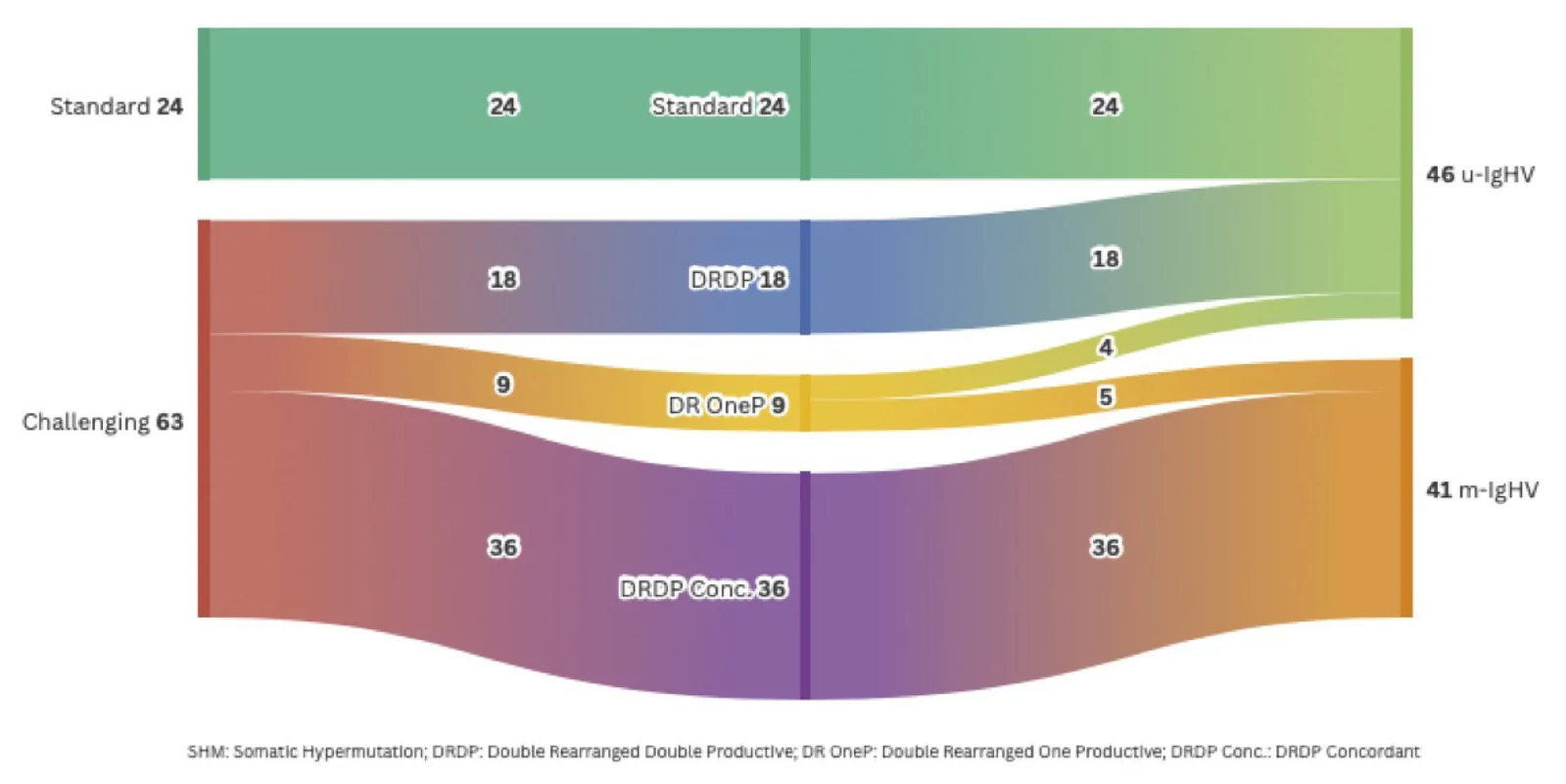
*IgHV* mutational status.

A total of 87 CLL patients were included in this study. The median age at diagnosis was 60 years (range: 29–84 years). Overall, 64% of the patients were male. While 10% of the patients presented with Rai stage III/IV disease, this proportion was 13% within the u-*IgHV* cohort (p = 0.036). A total of 24 patients (27.6%) were classified as standard cases. Double rearrangements (challenging cases) were detected in 63 patients (73%). As illustrated in Figure 2, detailed subanalysis of these challenging cases revealed three distinct patterns. The majority of the patients (57%) harbored two productive rearrangements that were both mutated (concordant double productive); these were classified as m-*IgHV*. One mutated and one unmutated clone were observed in 29% of the cases. In accordance with the ERIC guidelines for prognostic safety, these cases were classified as u-*IgHV*. The remaining cases showed double rearrangements, only one of which was productive. Based on the productive allele, 5 cases were classified as m-*IgHV* and 4 as u-*IgHV*. Consequently, combined with the 24 standard cases (all u-*IgHV*), the final cohort distribution included 46 (53%) u-*IgHV* patients and 41 (47%) m-*IgHV* patients. Among 83 patients analyzed for genetic abnormalities, 10 (12%) showed 17p deletions (Table 1). Extended cytogenetic data were available for a subset of 37 patients. Within this subgroup, isolated del(13q) was the most frequent abnormality (n = 7, 19%), followed by trisomy 12 (n = 3, 8%), *ATM* gene anomalies (n = 2, 5%), del(11q) (n = 1, 3%), and monosomy 8 (n = 1, 3%). Of the 87 patients, 47 (55 %) required treatment. Of those requiring treatment, 66% were in the u-*IgHV* group, a significantly higher rate compared to the m-*IgHV* group. The median time from diagnosis to initiation of treatment was 21.6 months (95% CI: 10.2–33.8). First-line treatments included chemoimmunotherapy (CIT) for 23 patients (49%) and targeted therapy (Bruton tyrosine kinase inhibitors or Bcl-2 antagonists) for 24 patients (51%). The median time from first-line to second-line treatment was 14.7 months (95% CI: 4.6–NA) in the u-*IgHV* group. These findings indicate that patients in the unmutated group required second-line treatment at earlier stages (Table 2).

### 3.2. *IgHV* mutational status and clinical outcomes

No significant differences were observed between the m-*IgHV* and u-*IgHV* groups regarding additional chromosomal abnormalities for del(11q), trisomy 12, monosomy 8, treatment indications based on iwCLL criteria, time to initial treatment, or Richter transformation. However, more patients in the unmutated group needed treatment and they had a greater frequency of 17p deletions (p = 0.048), while 13q deletion was less common in this group (p = 0.033). Additional findings included a shorter time from first-line to second-line treatment and a higher likelihood of ongoing treatment in the unmutated group compared to the mutated group (Table 2).

### 3.3. Survival

**Figure 3:**
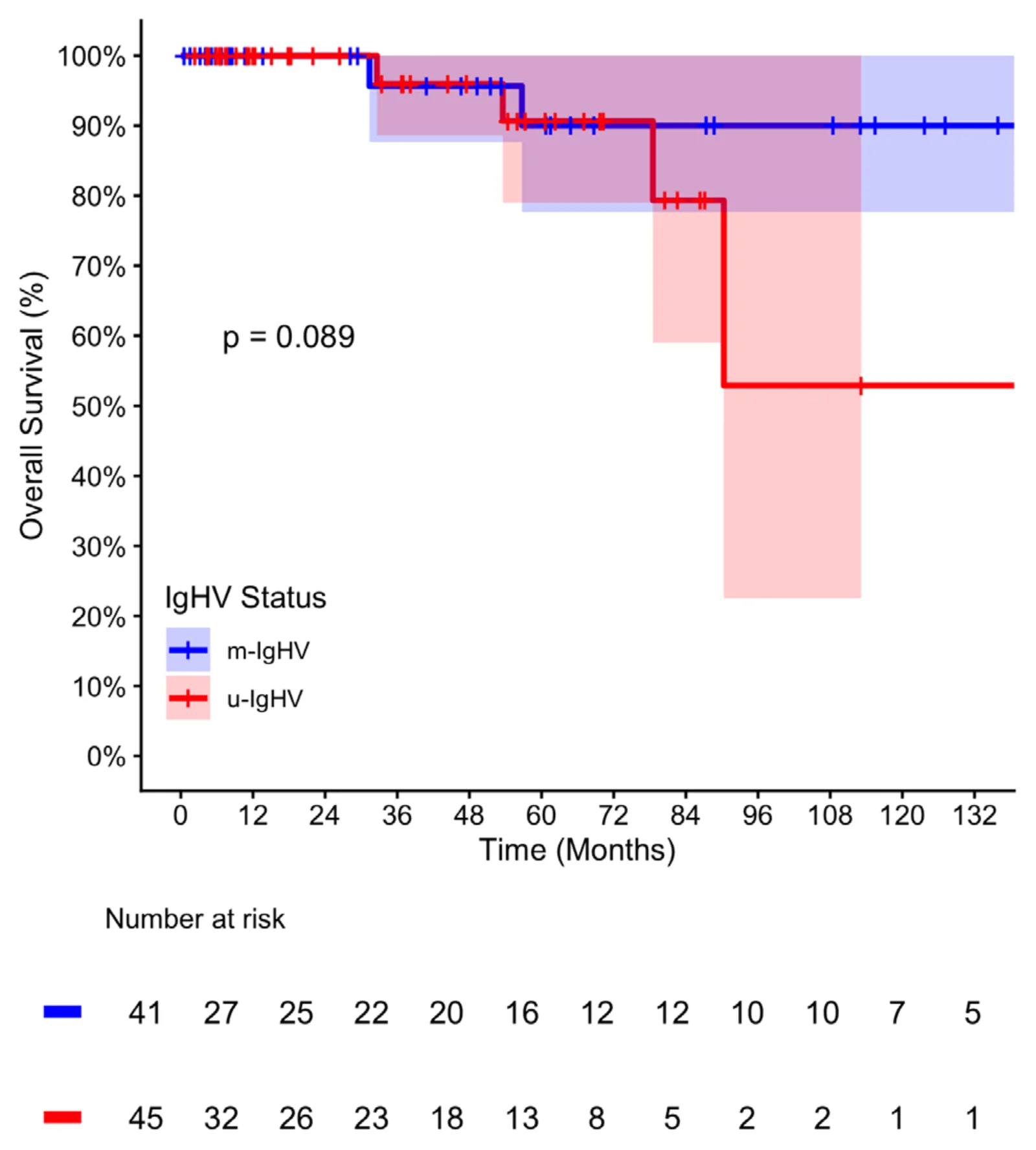
Overall survival analysis of patients according to *IGHV* mutation status.

The median follow-up was 37.5 months (3–203 months) and the OS rate was 91.9%. Thirty-seven patients (43%) were actively receiving treatment, with one receiving treatment for Richter transformation (1.2%). During follow-up, 80 patients (92%) survived and seven (8%) died. No statistically significant differences in OS were found between the m-*IgHV* and u-*IgHV* groups (95.1% and 88.9% respectively; p > 0.05) (Figure 3). The independent prognostic value of *IgHV* mutation status was further evaluated using a multivariate Cox proportional hazards models, incorporating del(17p) and CLL-IPI score for OS. The analysis revealed that u-*IgHV* status was associated with a nonsignificant trend toward increased mortality risk compared to m-*IgHV* status (HR: 3.304, 95% CI: 0.61–17.87, p = 0.16). In the same model, neither del(17p) (HR < 0.001, p = 0.99) nor CLL-IPI score (HR: 1.014, p = 0.96) were found to be independent predictors of survival. This is likely attributable to the limited number of mortality events (n = 7) in our cohort, which restricted the statistical power from reaching independent significance.

## 4. Discussion

In this retrospective analysis of the real-life data of CLL patients whose *IgHV* mutation statuses were determined using RNA-seq, a significant proportion of patients (73%) were classified as presenting challenging cases with double rearrangement. Notably, 63% of these cases exhibited double productive *IgHV* gene rearrangements without evidence of biclonality, as only a single light chain (either kappa or lambda) was detected. These findings suggest that RNA-based sequencing may facilitate the characterization of mutational complexity in CLL, particularly in cases where conventional methods are limited by technical constraints or ambiguous clonal patterns

The updated ERIC 2007 recommendations established a robust and comprehensive framework for the reliable interpretation of *IgHV* sequences, including the critical 98% identity threshold and early guidance for handling complex cases involving multiple rearrangements [10]. Building on this framework for interpreting complex profiles, the ERIC 2022 guidelines offer clear, practice-oriented guidance for “challenging” cases, particularly those with double productive rearrangements. They advocate a “biologically conservative” approach in discordant scenarios, where one allele is mutated and the other unmutated [3]. In our cohort, discordant cases were classified as u-*IgHV*. This strictly descriptive adherence to the guidelines minimizes the risk of misclassifying patients as low-risk (m-*IgHV*) in the presence of a potentially aggressive unmutated subclone, thereby helping to avoid undertreatment. Consistent with these recommendations, 52% of our patients were classified as u-*IgHV*, and according to published studies, the prevalence of u-*IgHV* is higher in patients with progressive disease (50%–60%) and even higher in those with relapse/refractory disease (70%–80%) [11].

The ERIC guidelines suggest that both complementary DNA (cDNA) and genomic DNA (gDNA) are suitable for determining *IgHV* mutational status. The standard approach involves PCR amplification of the expressed *IgHV* transcript, followed by Sanger sequencing and then comparison to germline sequences listed in immunoglobulin databases [12,13]. In a large study comprising 4154 cases, 435 cases (10.5%) were identified as having double rearrangement, considered to be diagnostically challenging, with a higher frequency observed when gDNA was used (14.8%) [9]. However, this method is prone to mispriming or complete amplification failure, particularly when SHM affects primer binding regions. In contrast, our findings demonstrated that the proportion of challenging cases increased to 73% with RNA sequencing, suggesting that RNA-based techniques may enhance the detection of challenging cases that might be missed by conventional methods.

RNA sequencing is a robust technique that not only reveals the underlying DNA sequence but also provides insight into both gene and isoform expression. It offers transcriptome data with high resolution and high dynamic range suitable for differential gene expression analyses, isoform discovery, and variant detection. In the context of CLL, RNA sequencing allows for precise determination of the *IgHV* gene sequence, including the complementarity determining region 3 (CDR3), and mutation status. While our study did not directly compare RNA-seq with DNA-based methods, our findings suggest that RNA-based classification is a feasible alternative that may help to address certain limitations of conventional techniques, such as the need to isolate a dominant clone, thereby offering valuable insight into the biological complexity of the disease. Moreover, this approach facilitates the interpretation of cases in which both mutated and unmutated clones coexist within the neoplastic B-cell population, addressing a significant challenge in CLL molecular profiling [6,14].

Accurate determination of *IgHV* mutation status is crucial, as it serves as a major prognostic biomarker for adjusting patient monitoring schedules and guiding treatment decisions when therapy is indicated. Furthermore, patients with u-*IgHV* are known to have a poorer prognosis. A metaanalysis revealed improved progression-free survival (PFS) in patients with m-*IgHV* compared to those with u-*IgHV*, with PFS ranging from 9.2 to 18.9 years and 1 to 5 years, respectively. Similarly, OS was 3.2 to 10 years in u-*IgHV* patients, being significantly shorter compared to m-*IgHV* patients [15]. Another study of 4135 patients provided real-life clinical outcomes stratified by *IgHV* mutational status over 3.5 years of follow-up, demonstrating that CLL patients with u-*IgHV* undergoing CIT had shorter OS and TTNT durations [16]. In our study, when OS was analyzed based on *IgHV* mutation status, we found no statistically significant difference between the two groups, contrary to expectations. These results could be explained by the relatively short follow-up period and the small sample size of our cohort. Additionally, the effectiveness of targeted therapies introduced with a better understanding of the pathogenesis may have contributed to overcoming the impact of negative prognostic markers.

In conclusion, CLL is a heterogeneous disease and *IgHV* mutation status remains a well-established prognostic marker. However, it does not fully account for the complexity of disease biology. With the increasing clinical application of *IgHV* mutation analysis, the identification of diagnostically challenging cases has become more frequent, likely due to the expanding depth of sequence data analyzed in recent cohorts. Further subclassification through BcR-Ig stereotypy studies has facilitated the recognition of biologically distinct subgroups within CLL, thereby enhancing our understanding of its diverse pathobiological features [17]. This study contributes valuable real-life data supporting the utility of a technique whose reliability has been highlighted in previous investigations.

## Figures and Tables

**Table 1:** Clinical characteristics.

Parameters	Overall (n = 87)	m-IgHV (n = 41)	u-IgHV (n = 46)	p

Age, median (min-max)	60 (29–84)	61 (29–81)	60 (39–84)	0.8
≥ 65 years, n (%)	30 (35)	14(34)	16 (36)	0.9
≥75 years, n (%)	10 (12)	5(12)	5 (11)	0.9

Gender, male, n (%)	55(64)	23 (56)	32 (71)	0.15

RAI stage, n (%)				0.2
0	12 (14)	7(17)	5 (11)	
1	41 (48)	19 (46)	22 (49)	
2	24 (28)	12 (29)	12(27)	
3	5 (6)	0	5 (11)	
4	4 (4)	3 (7)	1 (2)	

CLL-IPI				0.14
Low risk, n (%)	14 (16)	9 (22)	5 (11)	
Intermediate risk, n (%)	25 (29)	11 (27)	14 (31)	
High risk, n (%)	16 (19)	5 (12)	11 (24)	
Very high risk, n (%)	6 (7)	1 (2)	5 (11)	
Unknown, n (%)	25 (29)	15 (37)	10 (22)	

High-risk cytogenetic features (n:83)				0.048
del 17p	10 (12)	2(5)	8 (18)	

CLL-IPI: International Prognostic Index for Chronic Lymphocytic Leukemia.

**Table 2:** Treatment features and options.

Parameters	Overall (n=87)	m-IgHV (n=41)	u-IgHV (n=46)	p

Indication of treatment, n (%)	47 (55)	16 (34)	31 (66)	0.001

TTFT, months, median (95%Cl)	21.6 (10.2–33.8)	18.3 (3.7–123)	23.9 (7–43.9)	0.831

Treatment options in first line, n (%)				0.999
CIT	23 (49)	7 (44)	16 (52)	
Targeted therapy	24 (51)	9 (56)	15 (48)	

TTNT-1, months, median (95%Cl)	20.6 (6.3–64.8)	61.6 (35.9–NA)	14.7 (4.6–NA)	0.041

Treatment options in second line, n (%)				0.999
CIT	5 (31)	1 (20)	4 (36)	
Targeted therapy	11 (69)	4 (80)	7 (64)	

TTNT-2, months, median (95%Cl)	29.8 (28.9–NA)	66 (NA–NA)	29.4 (3.6–NA)	0.400

Treatment options in third line, n (%)				0.999
CIT	0	0	0	
Targeted therapy	5 (100)	1 (100)	4 (100)	

TTFT: time to first treatment; CIT: chemoimmunotherapy; TTNT-1: time to next treatment-1; TTNT-2: time to next treatment-2; NA: not reached. Upper bounds of the 95% confidence intervals were not reached in certain subanalyses due to the limited number of events during the follow-up period.
